# Sow Vaccination with a Protein Fragment against Virulent *Glaesserella (Haemophilus) parasuis* Modulates Immunity Traits in Their Offspring

**DOI:** 10.3390/vaccines9050534

**Published:** 2021-05-20

**Authors:** Sergi López-Serrano, Carlos Neila-Ibáñez, Mar Costa-Hurtado, Yasser Mahmmod, Jorge Martínez-Martínez, Iván José Galindo-Cardiel, Ayub Darji, Fernando Rodríguez, Marina Sibila, Virginia Aragon

**Affiliations:** 1 IRTA, Centre de Recerca en Sanitat Animal (CReSA, IRTA-UAB), Campus de la Universitat Autònoma de Barcelona, 08193 Bellaterra, Spain; sergi.lopez@irta.cat (S.L.-S.); Carlos.neila@irta.cat (C.N.-I.); mar.costahurtado@gmail.com (M.C.-H.); cdm141@alumni.ku.dk (Y.M.); Jorge.martinez@irta.cat (J.M.-M.); Ayub.darji@irta.cat (A.D.); fernando.rodriguez@irta.cat (F.R.); marina.sibila@irta.cat (M.S.); 2 Department of Animal Medicine, Faculty of Veterinary Medicine, Zagazig University, Zagazig 44511, Egypt; 3 Section of Veterinary Sciences, Health Sciences Division, Al Ain Men’s College, Higher Colleges of Technology, Al Ain 17155, United Arab Emirates; 4 OIE Collaborating Centre for the Research and Control of Emerging and Re-Emerging Swine Diseases in Europe (IRTA-CReSA), 08193 Bellaterra, Spain; 5 Departament de Sanitat i Anatomia Animals, Facultat de Veterinària, UAB, 08193 Bellaterra, Spain; 6 WorldPathol, Calle Ajedrea 20, Nave 22C, Parque Magnus-Polígono Empresarium, 50720 Zaragoza, Spain; ivan.galindo@worldpathol.com

**Keywords:** swine, bacteria, disease, vaccine, *Glaesserella parasuis*

## Abstract

*Glaesserella (Haemophilus) parasuis*, an early colonizer of the nasal cavity in piglets, is a highly heterogeneous species, comprising both commensal and virulent strains. Virulent *G. parasuis* strains can cause fibrinous polyserositis called Glässer’s disease. Colostrum is a source of passive immunity for young piglets. When vaccinating sows, protective antibodies are transferred to their offspring through the colostrum. Here, sow vaccination was performed with a protein fragment, F4, from the outer membrane trimeric autotransporters VtaAs exclusively found in virulent *G. parasuis*. Piglets were allowed to suckle for 3 weeks, following which a challenge with two virulent strains of *G. parasuis* was performed. A group of nonvaccinated sows and their piglets were included as a control. Antibodies against F4 were confirmed using ELISA in the vaccinated sows and their offspring before the *G. parasuis* challenge. Compared to the control group, F4-vaccination also resulted in an increased level of serum TGF-β both in vaccinated sows and in their offspring at early time points of life. After the challenge, a lower body temperature and a higher weight were observed in the group of piglets from vaccinated sows. One piglet from the non-vaccinated group succumbed to the infection, but no other significant differences in clinical signs were noticed. At necropsy, performed 2 weeks after the virulent challenge, the level of surfactant protein D (SP-D) in bronchoalveolar lavage was higher in the piglets from vaccinated sows. Vaccination did not inhibit the nasal colonization of the piglets by the challenge strains.

## 1. Introduction

*Glaesserella parasuis*, formerly known as *Haemophilus parasuis*, is a rod shaped, Gram-negative proteobacteria from the *Pasteurellaceae* family that is commonly found in the nasal microbiota of pigs. *G. parasuis* is a highly heterogeneous species, comprising commensal and virulent strains. Virulent strains can cause polyserositis, polyarthritis and meningitis, a pathological disorder called Glässer’s disease that is more prevalent in young piglets, especially in the nursery period.

The most commonly used treatment for Glässer’s disease and other bacterial diseases affecting piglets are antimicrobials. However, increasing concern about antimicrobial resistance arising from the livestock industry is promoting the research of alternative control tools, where vaccines have a relevant role [1].

*G. parasuis* is an extracellular pathogen, and the induction of opsonizing antibodies (for example, by vaccination) is essential for protection against disease [2,3]. Prevention of Glässer’s disease may be achieved with attenuated bacteria, inactivated bacterins and subunit vaccines [4]. However, commercial bacterins do not provide protection against all *G. parasuis* strains, promoting the search for alternative vaccines that could confer cross-protection among virulent strains of different serovars. Several vaccine candidates have been proposed in the literature [4], including virulence factors such as the virulence-associated trimeric autotransporters (VtaA), which are involved in phagocytosis resistance [5] and adhesion to extracellular matrix proteins [6]. In a previous study from our group, immunization with a combination of six VtaA proteins provided partial protection against a lethal infection with a virulent *G. parasuis* strain in colostrum deprived piglets [7]. A detailed study of the sequences of these proteins identified a peptide (F4) that was present in the VtaAs associated with virulent strains and was exposed on the bacterial surface [8], supporting its potential as a vaccine candidate.

In swine, maternal immunity is transferred to the litter via colostrum and progressively decreases with the age of the piglets, while the maturation of the immune system of the piglet is not yet complete [8,9]. Sow vaccination increases the level of colostrum antibodies and, subsequently, the period when maternal antibodies are maintained at a detectable level in their offspring [10,11]. For this reason, together with the short window available for piglet vaccination and their immature immune system, maternal immunization can be an option to prevent diseases early in life [12,13,14,15].

Here, we evaluate the effect on the offspring of sow vaccination using adjuvanted F4 peptide as an immunogen and the protection observed in the piglets after an intranasal challenge with two different virulent strains of *G. parasuis.*

## 2. Materials and Methods

### 2.1. Vaccine and Challenge Strains

The protein fragment F4, consisting of amino acids 701 to 874 from the trimeric autotransporter VtaA9 from *G. parasuis* [8], was used as an immunogen in combination with a carbomer-based adjuvant. Vaccine formulation was composed of the purified F4 fragment mixed with Carbopol 5984 EP Polymer (1:9) (Lubrizol, Cleveland, OH, USA) to achieve a final concentration of 100 µg/mL of F4 peptide (#F4 API VACSUIS, WorldPathol, Zaragoza, Spain).

*G. parasuis* strains Nagasaki (virulent reference strain serovar 5 [SV5]) and P555/04 (clinical isolate from pericardium, serovar 13 [SV13], provided by Dr. Zielinski from INTA-Argentina), were grown overnight on chocolate agar plates (Biomérieux, Marcy-L’Étoile, France) at 37 °C 5% CO_2_ and were used for the intranasal inoculation of piglets.

### 2.2. Animal Study

**Figure 1:**
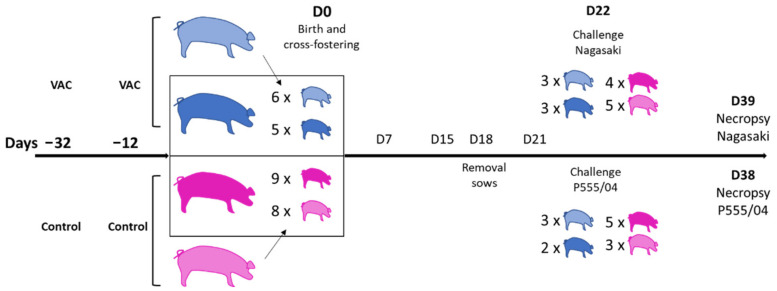
Schematic representation of the animal experiment. Two sows (in pale and dark blue) were vaccinated with VtaA fragment F4 at days −32 and −12 of farrowing, while two sows served as unvaccinated controls (in pale and dark pink). Al birth, D0, piglets (color indicates the sow of origin) were cross-fostered within each group and only one sow per group stayed in the study. Later, piglets were split in groups for inoculation at day 22 with two different serovars of *G. parasuis*: Nagasaki strain (SV5) represented in the upper part of the graph; and P555/04 (SV13) in the lower part. After 2 weeks, necropsies and final sampling were performed. Sampling points during the experiment were days 7, 15 and 21.

Animal experimentation was performed in the BSL3 facilities of IRTA-CReSA (Bellaterra, Spain) following proper veterinary practices, in accordance with European (Directive 2010/63/EU) and Spanish (Real Decreto 53/2013) regulation and with the approval of the Ethics Commission in Animal Experimentation of the Generalitat de Catalunya (Protocol number 9211). The experimental procedure is represented in Figure 1. Four pregnant sows were selected on the basis of low antibody levels against *G. parasuis* using the commercial ELISA Ingezim–Haemophilus (Ingenasa, Madrid, Spain). Two sows were vaccinated at 32 and 12 days before farrowing with 2.5 mL of the F4 vaccine, while the other two sows remained unvaccinated as controls. Serum samples were taken from the sows at each time of vaccination and at delivery. In addition, colostrum samples were taken at delivery. After birth, piglets suckled from their mother for 1 day and then the litters were cross-fostered within each experimental group. One vaccinated sow (with five of its own piglets and six from the other vaccinated sow) and one nonvaccinated control sow (with nine of its own piglets and eight from the other control sow) remained in the study. Sows were removed from the study at day 18 (D18). At 1, 2 and 3 weeks of age, nasal samples were taken from the piglets for *G. parasuis* detection. At 1, 2 and 3 weeks, serum samples were also obtained. One day later (D22), piglets were divided into two groups for the challenge, with piglets from the different biological sows in each group. Thus, nine piglets from nonvaccinated sows and six from vaccinated sows were intranasally inoculated using an atomizer with 6 × 10^9^ colony forming units (CFU) of Nagasaki (SV5) with a total inoculation volume of 1.5 mL per piglet, while eight piglets from nonvaccinated sows and five from vaccinated sows were intranasally inoculated with 3 × 10^9^ CFU of P555/04 (SV13) in 1.5 mL (Figure 1). After 16 (P555/04 group) and 17 (Nagasaki group) days of challenge, final sampling and necropsies were performed for pathological examination and samples from different tissues were taken for analysis. Serum and bronchoalveolar lavage fluid (BALF) were also taken for immunological analysis.

### 2.3. Clinical Signs and Pathological Assessment

Rectal temperatures, weight and clinical signs (particularly those compatible with Glässer’s disease) were evaluated after the challenge with *G. parasuis*. Fever was considered to be a rectal temperature above 40 °C. Clinical signs were scored daily for each piglet, and later, a global clinical score was used for analysis: 0 = no symptoms; 1 = clinical signs on 1 day; 2 = clinical signs on more than 1 day; 3 = severe clinical signs (euthanasia).

Gross lesions were assessed in necropsy and the presence of *G. parasuis* in different organs was determined by culturing samples from body cavities (pericardic, thoracic and abdominal), four joints and blood.

Samples from the lung, trachea and nasal turbinate were taken for histopathological examination. Formalin fixed tissues were embedded in paraffin and 3–5 µm sections were cut for haematoxylin-eosin staining. Assessment of lymphoplasmacytic inflammation was scored as: 0, absence; 1, mild; 2, moderate; 3, severe.

### 2.4. DNA Extraction and G. parasuis Detection by PCR

Nasal swabs were resuspended in 500 µL of PBS and 200 µL of the suspensions were processed using the Nucleospin Blood kit (Macherey-Nagel, Düren, Germany) according to the manufacturer’s instructions. For pure bacterial cultures, DNA was extracted using a Chelex based Instagene Matrix (Bio-Rad Laboratories, Hercules, CA, USA) following the manufacturer’s instructions. *G. parasuis* was detected and differentiated into virulent and nonvirulent using a specific *vtaA* leader sequence PCR (LS-PCR) [16]. In some cases, additional molecular serotyping PCR was performed for serovars 5/12 and 13 [17]. Four µL of the DNA extracted from the nasal swabs or two µL of the purified DNA from pure cultures were used as template for the reactions. DNA from strains Nagasaki (serovar 5, virulent), P555/04 (serovar 13, virulent) and SW114 (nonvirulent) were included as controls for the PCRs. Primers used in the PCRs can be found in Table S1.

### 2.5. Antibody Response

Antibodies were measured using ELISA as follows: High binding plates were coated overnight at 4 °C with F4 protein (500 ng/well) in a carbonate–bicarbonate buffer. After washing, wells were blocked with 1% casein in phosphate-buffered saline (PBS) with 0.05% Tween 20 (PBS-Tw). Sera and colostrum were diluted 1:1000 in blocking solution and BALF samples were used undiluted. After an incubation of 1h at 37 °C, a goat antiporcine IgG HRP-conjugated antibody (Sigma-Aldrich, Madrid, Spain) was used for IgG detection at a 1:10,000 dilution in blocking solution. For IgA detection in BALF, samples were tested undiluted and detected with an HRP-conjugated goat anti-porcine IgA antibody (AbD Serotec, Oxford, UK) diluted 1:10,000. Finally, positive reactions were developed using the 3,3,3,5-tetramethylbenzidine (TMB) substrate (Sigma-Aldrich, Madrid, Spain) and the reaction was stopped with 1N sulfuric acid. Plates were then read in a Power Wave XS spectrophotometer (Biotech, Winooski, VT, USA) at 450 nm.

### 2.6. Interleukin Determination

Serum levels of IL-8, IL-10, TNF-α and TGF-β were evaluated via ELISA using different products. Two matched antibody pairs for swine IL-10 and TNF-α from Kingfisher Biotech (Saint Paul, MN, USA) and an ELISA kit from the same manufacturer for porcine IL-8 were used. TGF-β was evaluated using a TGF-β Human Matched Antibody Pair kit from Invitrogen (Carlsbad, CA, USA), following the manufacturer’s instructions in all cases.

### 2.7. Surfactant Protein D (SP-D) Monoclonal Antibodies Production and SP-D Determination by ELISA

Surfactant protein D (SP-D) was determined via an in-house sandwich ELISA using a monoclonal antibody pair (capture and detection) from hybridoma supernatants, whose production was performed as follows. Briefly, lung SP-D was extracted and purified from the BALF of healthy piglets following previously described protocol [18]. Hybridomas were generated by immunizing BALB/c mice with the purified SP-D using standard procedures described before [19] and with the approval of the Ethics Commission in Animal Experimentation from the Generalitat de Catalunya (Approved protocol 5767). Two monoclonal antibodies were selected according to their strong signal against the SP-D antigen via indirect ELISA, and their isotype was determined using a mouse typer isotyping kit (Bio-Rad, Hercules, CA, USA), following the manufacturer’s indications. Selected clones were R-133 (IgG2b) (used as a capture antibody) and R-123 (IgG1) (used for detection).

For ELISA, high binding 96 well plates were coated with 50 µL of the capture antibody hybridoma supernatant R-133 diluted 1:1 in carbonate bicarbonate buffer overnight at 4 °C. After coating, plates were washed and blocked with blocking solution as above for 1h at 37 °C. After an incubation of 50 µL of undiluted BALF for 1 h at 37 °C, the plates were washed, and a detection antibody mixture, which consisted of a mixture 1:1 of the hybridoma supernatant R-123 with blocking buffer, was added to the wells. After incubation for 1 h at 37 °C and washes, ELISA plates were finally incubated for 1 h at 37 °C with an HRP-conjugated goat anti-mouse IgG1 antibody (Invitrogen, Carlsbad, CA, USA) diluted 1:1000. Finally, reaction was developed with TMB for 5–10 min, and stopped with 1N sulfuric acid before reading at 450 nm.

### 2.8. Statistical Analysis and Modelling

At first, the collected data were screened for unlikely or missing values before running any valid statistical analysis. No data were excluded on this basis. Subsequently, a descriptive statistical analysis was performed to the potential predictors related to piglets and sows in the experiment with the main variables of interest: clinical signs, biological sows, sow vaccination, challenge strain and colonization with virulent strains of *G. parasuis* (GP.VIR) or nonvirulent strains (GP.NVIR) at the end of the study.

Different statistical models of multilevel multivariable linear regression with repeated measurements were run for the following continuous outcome variables: IL-8, IL-10, TGF-β, antibody levels against F4, SP-D levels in BALF, rectal temperature, weight after challenge, IgG against F4 in BALF, total IgG in BALF and ratio of α-F4 IgG/total IgG. Sow-ID was accounted as a random effect in the different models. The outcome variable that showed skew pattern was, therefore, transformed by taking their natural logarithm or log10.

A univariable model analysis was carried out to test the unconditional associations between dependents and different independent variables of interest. Only independent variables with *p* ≤ 0.25 in this initial screening were included in multivariable linear regression models [20]. Before proceeding with building our multivariable model analysis, we checked for correlations between the retained independent variables. If two variables were highly correlated (R > 0.8), only the one with the lower *p* value in the unconditional associations was retained.

The significant predictors from the univariable analysis were then offered to a multivariable model, and a manual backward elimination procedure was used to the least significant variable (*p* > 0.05) if their exclusion from the model did not result in a greater than 30% change (confounding effect) in the effects of the remaining variables in the model [20].

The generated final model included only variables with a *p* value ≤ 0.05. In this model, two-way interactions between the remaining significant variables were investigated and were retained if significant. The *p* value and regression coefficient (b) with a 95% confidence interval (95% CI) were recorded for each variable. In all statistical analyses, the results were considered to be significant with a *p* ≤ 0.05.

All statistical analyses were conducted using R version 3.3.3 (R Core Team, 2015).

## 3. Results

### 3.1. Sow Vaccination with F4 Induces Specific Antibodies and TGF-β in the Offspring

**Figure 2:**
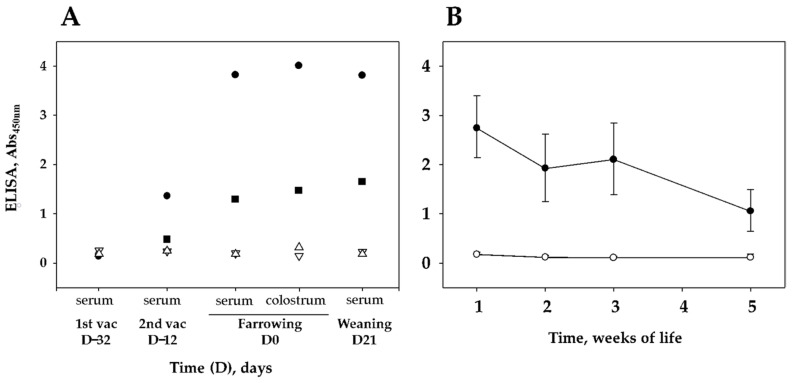
Levels of IgG against F4 in samples from sows (**A**) and their piglets (**B**). (**A**), Two sows were vaccinated (black symbols) with F4 at 4 (day −32) and 2 (day −12) weeks before farrowing. Two sows remained unvaccinated as control (white symbols). Serum samples at vaccination times, farrowing (D0) and at weaning (3 weeks after farrowing) (as well as colostrum samples) were tested for F4 antibodies using ELISA. (**B**), Serum samples from piglets nursed by vaccinated (black circles) or nonvaccinated (white circles) sows taken at 1, 2, 3 and 5 weeks of life were tested for F4 antibodies by ELISA. At 22 days of life, piglets were challenged with *G. parasuis*. The average absorbance in the ELISA is shown in the graph. Error bars represent the standard deviation. Individual piglet results can be found in Figure S1.

The response to vaccination in sows was evaluated in colostrum and serum at different times postvaccination (Figure 2A). It is noteworthy that one of the vaccinated sows showed higher level of antibodies against F4 than did the other sow. As expected, sera from piglets reflected the vaccination of the sows, with higher level of antibodies against F4 in piglets nursed by vaccinated sows than in piglets from unvaccinated sows. Antibody levels showed a decreasing tendency during the first two weeks of life and a stabilizing tendency between the second and the third week of life, at the time of the *G. parasuis* challenges (Figure 2B). Interestingly, the level of F4 antibodies dramatically decreased immediately after the G. *parasuis* challenges until the end of the experiment (Figure 2B), most probably reflecting the specific sequestering of F4-specifcic antibodies by the bacteria. As expected, the multivariable statistical model showed a significative association of the level of F4 antibodies (Log10 of ELISA absorbance) with sow vaccination (*p* = 1.53 × 10^−11^; Table 1).

**Figure 3:**
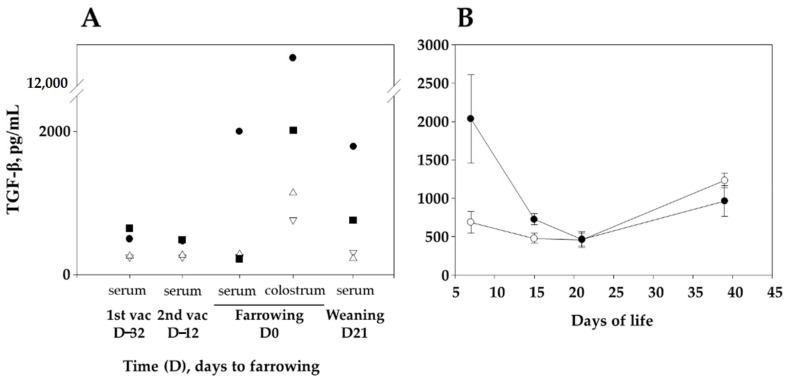
Levels of TGF-β in samples from sows (**A**) and their piglets (**B**). The concentration of TGF-β in serum samples taken in sows and piglets at vaccination times, farrowing and weaning (3 weeks after farrowing) (as well as in colostrum sample), was measured using ELISA. (**A**), Two sows (black symbols) were vaccinated with F4 at 4 (day-32) and 2 (day-12) weeks before farrowing. The other two sows remained unvaccinated as control (white symbols). (**B**), Serum samples from piglets nursed by vaccinated (black circles) or nonvaccinated (white circles) sows were taken at 1, 2, 3 and 5 weeks of life. At 22 days of life, piglets were challenged with *G. parasuis*. The average concentration of serum TGF-β in each group is shown in the graph. Error bars are the standard deviation. Individual piglet results can be found in Figure S2.

Vaccinated sows had higher TGF-β levels in serum and colostrum at the time of farrowing (Figure 3A), with each one responding differently. In piglets, TGF-β levels in sera were higher in animals from vaccinated sows than in those from nonvaccinated ones at 7 and 15 days of life (two-tailed Student *t* test, *p* < 0.0001 for both days), but values stabilized and were similar in both groups of piglets just before the challenge at D21 (Figure 3B). In addition, a correlation between the level of F4-antibodies and TGF-β in the piglets at 7 days of life was observed (R = 0.68). To study the association between TGF-β levels and other parameters, values were log-transformed to achieve normal distribution. Statistical multivariable model confirmed the significant association of sow vaccination with TGF-β levels in piglets (*p* = 1.281 × 10^−5^). Piglets from vaccinated sows had a higher log level of TGF-β by 0.498 (95% CI 0.241–0.757) in comparison to piglets from nonvaccinated sows.

**Figure 4:**
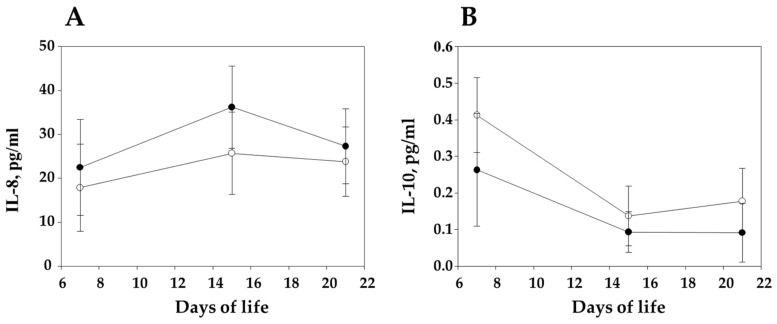
Levels of IL-8 (**A**) and Il-10 (**B**) in sera from piglets born to vaccinated and nonvaccinated sows. Serum samples were taken at 7, 15 and 21 days of life. Empty dots represent the piglets from nonvaccinated sows, whereas black dots represent piglets nursed by vaccinated ones. Individual piglet results can be found in Figure S3A,B.

Although the levels of IL-8 in sera were low through the study, log IL-8 levels in the piglets were also associated with sow vaccination (Figure 4A and Table 2), and, in addition, it was negatively correlated with the colonization of the piglets with *G. parasuis* virulent strains at the end of the study (Table 2). The opposite tendency was observed with IL-10 and vaccination (Figure 4B), since piglets from vaccinated sows had lower log IL-10 levels by −0.093 in comparison to piglets from nonvaccinated sows. Again, the levels of IL-10 through the study were kept low. TNF-α in serum displayed values below the detection limit of the technique in all the samples.

### 3.2. Clinical Signs and Pathological Findings after Virulent G. parasuis Challenge

Piglets were weaned and then distributed into two groups and challenged at D22 with two different virulent *G. parasuis* strains (Figure 1). Rectal temperatures were taken after the infection with the virulent *G. parasuis*. The final multivariable model showed a significant association between the body temperature of the piglets and sow vaccination. We found that piglets nursed by vaccinated sows had lower rectal temperature after challenge by −0.095 in comparison to the piglets of nonvaccinated sows (Table 3).

After challenge, only one piglet infected with the Nagasaki strain from the unvaccinated sow group succumbed to the infection and showed severe lesions of Glässer’s disease, presenting fibrin aggregates in the pericardium, thoracic and abdominal cavities. The rest of the piglets showed mild clinical signs. Regarding the strain used in the challenge, more animals infected with Nagasaki presented clinical signs (9/15) than those infected with P555/04 (5/13), but no significant differences between piglets from vaccinated or nonvaccinated sows were detected within the two challenge groups in terms of disease outcome. Unexpectedly, the antibody levels against F4 were significantly associated in piglets with clinical signs (*p* = 0.0083; Table 1). However, a close examination of the groups showed that the group challenged with Nagasaki (which caused more clinical signs due to its higher virulence) also contained more piglets with higher level of antibodies before the challenge. This may at least in part explain this unexpected observation.

Microscopical observation of hematoxylin/eosin-stained nasal turbinates showed that animals infected with the Nagasaki strain presented a higher inflammation score than animals infected with P555/04 (Fisher’s exact test, *p* = 0.006). However, no significant differences were found between piglets from vaccinated or nonvaccinated sows.

### 3.3. Weight Gain after Challenge

The weight of the animals was monitored after challenge, and piglets from vaccinated sows gained significantly more average weight (by 1.159 units) than piglets from unvaccinated ones (Table 4). Our findings also showed that the colonization of the nasal mucosa of piglets with nonvirulent *G. parasuis* at the time of necropsies (GP.NVIR on Table 4) was associated with an increase in their average weight by 1.148 units in comparison to piglets without this colonization.

### 3.4. Piglets from Vaccinated Sows Showed Higher Titres of F4 Antibodies and SP-D in BALF

**Figure 5:**
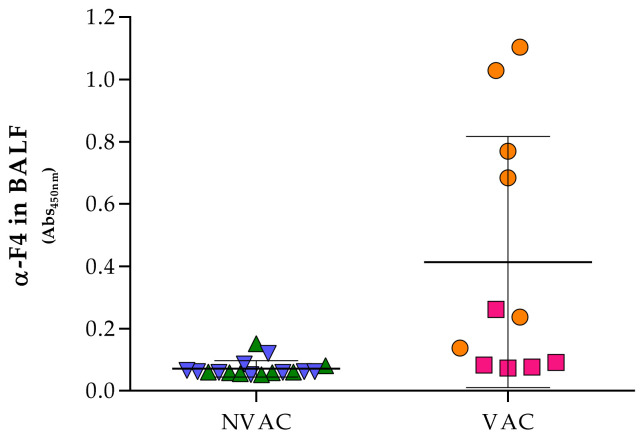
Levels of IgG against F4 in bronchoalveolar lavage fluid (BALF). Piglets born to vaccinated (VAC, pink and orange by biological sow) or nonvaccinated (NVAC, blue and green by biological sow) sows were challenged with *G. parasuis* at 22 days of life and 2 weeks later were euthanized. At that time, BALF samples were taken and F4 antibodies were measured by ELISA. The individual value for each piglet and the average absorbance in the ELISA (horizontal lines) are shown in the graph. Error bars are the standard deviation.

BALF samples were obtained at necropsy, two weeks after the challenge with the virulent *G. parasuis* strains. Specific anti-F4 IgG were detected at higher levels in the BALF from piglets born to vaccinated sows than from those born to nonvaccinated ones (two-tailed Student *t* test, *p* = 0.0016; Figure 5). For the multivariable model, the ratio of specific F4 IgG to the total IgGs in BALF was calculated and transformed into log10 in order to achieve its normalization. The model showed a direct correlation of this ratio with sow vaccination (*p* = 1.89 × 10^−^^5^; Table 5) and an additional association with the challenge strain, with piglets challenged with P555/04 having a lesser level of F4 antibodies/total IgG by −0.308 than those challenged with Nagasaki (Table 5).

**Figure 6:**
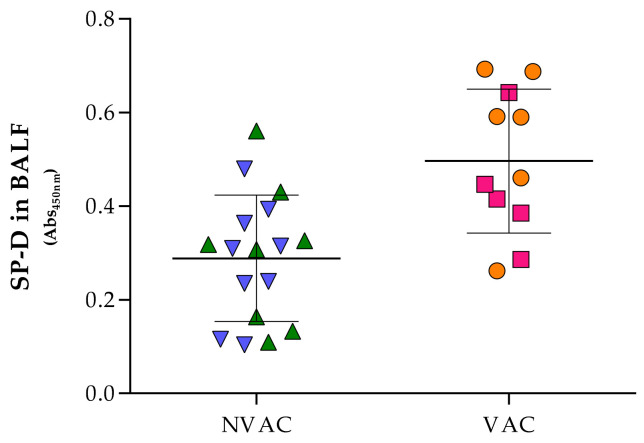
Levels of surfactant protein D (SP-D) in bronchoalveolar lavage fluid (BALF). Piglets born to vaccinated (VAC, pink and orange by biological sow) or nonvaccinated (NVAC, blue and green by biological sow) sows were challenged with *G. parasuis* at 22 days of life and 2 weeks later were euthanized. At that time, BALF samples were taken and SP-D was measured by a capture ELISA. The individual value for each piglet and the average absorbance in the ELISA (horizontal lines) are shown in the graph. Error bars are the standard deviation.

SP-D detection in BALF obtained at necropsy two weeks after the challenge showed that piglets from vaccinated sows had higher levels of this protein in BALF than those from nonvaccinated sows (two-tailed Student *t* test, *p* = 0.0009; Figure 6). Multivariable statistical model revealed that sow vaccination and challenge strain were significantly associated with SP-D levels in BALF (Table 6). Piglets from vaccinated sows had higher SP-D levels in BALF by 0.21 in comparison to piglets from nonvaccinated sows. P555/04 strain induced lower levels of SP-D in BALF by −0.15 in comparison to the Nagasaki strain (Table 6).

### 3.5. Sow Vaccination Did Not Inhibit Nasal Colonization of the Offspring by G. parasuis

To assess the colonization of the piglets by the challenge strains, nasal swabs were analyzed using PCR. Using a virulence-specific PCR based on the leader sequence of *vtaA* (LS-PCR), *G. parasuis* virulent strains were not detected in the nasal cavity of the piglets before the challenge, but they were detected at the time of necropsy (Table 7). When we used serovar specific PCRs to detect the challenge strains, serovar 5 was detected more frequently in the nose of nonvaccinated animals (8/9) than in vaccinated ones (3/6), but this difference was not statistically significant. In the piglets challenged with the P555/04 strain, no differences were detected in the presence of serovar 13 in the nose of the vaccinated or nonvaccinated piglets (Table 7). In addition, nonvirulent strains were detected at the time of necropsy in most of the piglets in all the experimental groups.

## 4. Discussion

Maternal-derived immunity through the colostrum is essential for the protection of young piglets, and sow vaccination is a way to increase maternal-derived antibody levels in the piglets, which may help preventing infectious diseases in the postweaning period. In our study, sow vaccination with the VtaA fragment F4 resulted in increased specific antibody and high TGF-β levels in the sera of sows and their offspring. After the challenge with virulent *G. parasuis*, piglets from vaccinated sows presented milder clinical signs and increased SP-D and anti-F4 IgG antibody levels in their lungs. Besides these parameters, piglets from vaccinated sows gained more weight after the infection than animals from unvaccinated sows, reflecting a healthier status. These results confirm the importance of maternal immunity in piglets, where sow vaccination may represent a good alternative in contrast to piglet immunization for the control of early life diseases, since the immature piglet immune system could benefit from their mother’s immunity transfer.

Sow sera and colostrum contained high anti-F4 IgG levels in response to the vaccine, which were subsequently transferred to their offspring. It is reported that the transference of maternal antibodies and other immune components can protect newborn piglets against pathogens during lactation until their own immune system matures [21]. Together with specific antibodies, an increase of TGF-β in piglets born to vaccinated sows was observed in our study. These outcomes are in line with those reported in human [22], where an upregulation of TGF-β in the mucosa of the upper airways was detected in neonates from mothers vaccinated with Influenza pdm09 H1N1 adjuvanted vaccine during the pregnancy. Likewise, TGF-β levels were increased in vaccinated sow sera and especially more markedly in colostrum. According to the literature, TGF-β represents one of the most important cytokines transferred by lactation and helps in the maturation of the gastrointestinal tract of the suckling animals helping in IgA class-switching [23,24,25]. A similar trend of maternal transference, although much more diminished than that of TGF-β, was also observed in our experiment with IL-10. These two cytokines have among its pleiotropic functions an immune regulation activity and are principally secreted by T-regulatory cells. This immune suppression in such young stage plays an important role in preventing the exacerbation of the immune system, when the animals begin their contact with the environment and their mucosae begin to be colonized [26,27]. Interestingly, an upregulated gene expression of TGF-β was observed in the lungs of animals that were fully-resistant to Glässer’s disease in comparison to susceptible ones [28]. In that study the authors relate this upregulation with early tissue-repair and antibacterial activity. Moreover, an in vitro experiment with PK-15 cells inoculated with virulent *G. parasuis* showed that TGF-β may also play an important protective function by inducing the expression of fibronectin, collagen and integrins necessary to preserve the integrity of the tissue and therefore prevent the invasion of the pathogen [29]. Glässer’s disease is an inflammatory illness where TGF-β may help in controlling an excessive inflammatory response to the infection, which may facilitate bacterial clearance without the deleterious effects of an excessive activation that causes the typical lesions of polyserositis of the disease. Induction of TGF-β was not observed in sows vaccinated with vaccines against *Mycoplasma hyopneumoniae* or porcine circovirus type 2 (unpublished results). However, an increase in TGF-β was detected in piglets vaccinated with F4 protein adjuvanted with Alum in comparison with its corresponding controls and with other adjuvants like carbomer and complete Freund (unpublished results). For these reasons, we consider that the mechanism by which TGF-β increases after vaccination with F4 deserves further studies, as we cannot rule out a role of the adjuvant in the stimulation or a direct induction by the F4 protein, as it has been observed with other bacterial adhesins with anti-inflammatory activity [30].

To our knowledge, this is the first report showing that maternal vaccination significantly increased the levels of SP-D in the piglets’ lungs. SP-D is a collectin, one of the first humoral defenses against pathogens. SP-D is secreted by different epithelia throughout the body, specially abundant in digestive and respiratory tracts, with functions comprising from the opsonization and neutralization of pathogens to modulation of the immune response [31]. However, the link between sow vaccination and SP-D production in piglets’ lungs is not well understood and further research would be needed. In this study we also found a correlation between sow vaccination and F4 antibodies in the BALF of the offspring, which can play a role in bacterial opsonization to allow clearing by alveolar macrophages [2].

Virulent *G. parasuis* strains colonize the upper respiratory tract before progressing into the lung, where innate immunity mechanisms survive [2,32]. Our experiment showed that both strains used for challenge were able to colonize the nasal cavity of the piglets with different severity outcomes afterwards. Animals challenged with the Nagasaki strain showed more inflammation in the nasal cavity (via inoculation) and a greater production of SP-D and lung anti-F4 antibodies in comparison to P555/04, which we attribute to the higher virulence of Nagasaki. Although we observed one fatal case of Glässer’s disease in the nonvaccinated animal group challenged with the Nagasaki strain, we could not fully evaluate the protection ability of the vaccine. Clinical signs were generally mild, probably due to the use of sow-reared piglets [33]. On the other hand, nasal colonization by virulent strains seemed to be reduced by vaccination, although not significantly, while nonvirulent strains were found in most of the piglets included in the experiment. This result may indicate that sow vaccination with F4, a protein fragment only found in virulent *G. parasuis* strains, does not affect those commensal nonvirulent strains that colonize the upper respiratory tract and are unable to reach the lung.

## 5. Conclusions

The vaccination of sows with VtaA fragment F4 had a clear impact on the levels of specific antibodies, TGF-β and lung SP-D in their offspring. After the experimental challenge using two different virulent strains of *G. parasuis*, piglets from vaccinated sows suffered milder clinical signs and gained more weight than controls. These results indicate that maternal vaccination could contribute to control Glässer’s disease in young piglets, a strategy that deserves further research.

## Figures and Tables

**Table 1:** Final model with the association of the log_10_ transformed ELISA values against F4 and predictor variables using generalized additive model.

Variable	Level	Estimate	95% CI	*p* Value
Sow vaccine	Nonvaccinated	Ref	Ref	1.53 × 10^−11^
	Vaccinated	0.826	0.713–0.941	
Clinical signs	Absence	Ref	Ref	0.0083
	Presence	0.105	0.029–0.181	

**Table 2:** Final model with the association of log10 IL-8 and predictor variables using multivariable mixed linear regression with time as a random effect. GP.VIR: colonization of the nasal cavity of the piglets with virulent *G. parasuis* strains at the end of the study.

Variable	Level	Estimate	95% CI	*p* Value
Sow vaccine	Nonvaccinated	Ref	Ref	0.0463
	Vaccinated	8.370	0.269–16.469	
GP.VIR	Absence	Ref	Ref	0.0258
	Presence	−9.825	(−18.295)–(−1.354)	

**Table 3:** Final model with the association of body temperature of piglets and other predictor variables using multivariable linear regression with repeat measurements while accounting for sow identity as random effect.

Variable	Level	Estimate	95% CI	*p* Value
Sow vaccine	Nonvaccinated	Ref	Ref	0.0114
	Vaccinated	−0.095	(−0.168)–(−0.021)	
GP.VIR	Absence	Ref	Ref	0.324
	Presence	0.024	(−0.036)–0.083	
Clinical signs	Absence	Ref	Ref	0.0702
	Presence	−0.045	(−0.092)–0.003	

**Table 4:** Final model with the association of body weight of piglets after challenge and predictor variables using multivariable linear regression with repeat measurements while accounting for sow identity as random effect.

Variable	Level	Estimate	95% CI	*p* Value
Sow vaccine	Nonvaccinated	Ref	Ref	0.00541
	Vaccinated	1.1597	0.363–1.957	
GP.NVIR	Absence	Ref	Ref	0.039015
	Presence	1.1481	0.079–2.221	

**Table 5:** Final model with the association of log10 of the ratio of specific F4 IgG to total IgG in BALF and predictor variables using multivariable linear regression with repeat measurements while accounting for sow identity as random effect. BALF samples were taken from piglets after necropsy.

Variable	Level	Estimate	95% CI	*p* Value
Sow vaccine	Nonvaccinated	Ref	Ref	1.89 × 10^−^^5^
	Vaccinated	0.666	0.406–0.927	
Challenge strain	Nagasaki	Ref	Ref	0.0165
	P555/04	−0.308	(−0.564)–(−0.053)	

**Table 6:** The final model with the association of SP-D level BALF in piglets and predictor variables using multivariable linear regression with repeat measurements while accounting for sow identity as random effect.

Variable	Level	Estimate	95% CI	*p* Value
Sow vaccination	Nonvaccinated	Ref	Ref	0.00022
	Vaccinated	0.21	0.11–0.31	
Challenge strain	Nagasaki	Ref	Ref	0.0054
	P555/04	−0.15	(−0.25)–(−0.046)	

**Table 7:** Detection of virulent (VIR) and nonvirulent (NVIR) *Glaesserella parasuis* strains in nasal swabs by LS-PCR and serovar PCR at different times in piglets born to vaccinated or nonvaccinated sows after an intranasal challenge with two virulent strains of *G. parasuis* (serovar SV5 Nagasaki and serovar SV13 P555/04) at 22 days of life.

		D7		D39		D39	
Challenge Strain	Piglets from Sows	VIR	NVIR	VIR	NVIR	SV 5/12	SV 13
Nagasaki	Vaccinated	0/6	3/6	4/6	4/6	3/6	
			(50%)	(67%)	(67%)	(50%)	
	Nonvaccinated	0/6					
			6/9	8/9	8/9	8/9	
			(67%)	(89%)	(89%)	(88.9%)	
P555/04	Vaccinated	0/5					
			2/5	4/5	4/5		4/5
			(40%)	(80%)	(80%)		(80%)
	Nonvaccinated	0/8					
			4/8	4/8	7/8		4/8
			(50%)	(50%)	(88%)		(50%)

## Data Availability

Not applicable.

## Supplementary Materials

The following are available online at https://www.mdpi.com/article/10.3390/vaccines9050534/s1, Figure S1: Levels of IgG against F4 in samples from individual piglets. Piglets from two vaccinated sows (vaccinated with F4 at 4 and 2 weeks before farrowing; pink and orange symbols and lines) and from two nonvaccinated sows were studied (blue and green symbols and lines). Serum samples from the piglets were taken at 1, 2, 3 and 5 weeks of life and tested for F4 antibodies by ELISA. At 22 days of life, piglets were challenged with *G. parasuis*. The average absorbance in the ELISA of the piglets from each biological sow is shown in the graph (lines), Figure S2. Levels of TGF-β in samples from individual piglets. Piglets from two vaccinated sows (vaccinated with F4 at 4 and 2 weeks before farrowing; pink and orange symbols and lines) and from two nonvaccinated sows were studied (blue and green symbols and lines). Serum samples from the piglets were taken at 1, 2, 3 and 5 weeks of life and the level of TGF-β was determined. At 22 days of life, piglets were challenged with *G. parasuis*. The average quantity of TGF-β of the piglets from each biological sow is shown in the graph (lines), Figure S3. Levels of IL-8 (A) and IL-10 (B) in samples from individual piglets. Piglets from two vaccinated sows (vaccinated with F4 at 4 and 2 weeks before farrowing (pink and orange symbols and lines) and from two nonvaccinated sows were studied (blue and green symbols and lines). Serum samples from the piglets were taken at 7, 15 and 21 days of life and the levels of IL-8 (A) and IL-10 (B) were determined. The average quantity of IL-8 (A) and IL-10 (B) of the piglets from each biological sow is shown in the graph (lines), Table S1: Primers used in the study for detection of *Glaesserella parasuis*.
