# Supplementary material for: Sow Vaccination with a Protein Fragment against Virulent Glaesserella (Haemophilus) parasuis Modulates Immunity Traits in Their Offspring
Source: Vaccines (Basel). 2021 May 20;9(5):534. doi: 10.3390/vaccines9050534 (PMC8160652; doi:10.3390/vaccines9050534)
Supplement: Supplementary file 1 [file vaccines-09-00534-s001.zip › vaccines-1212851-supplementary.pdf]

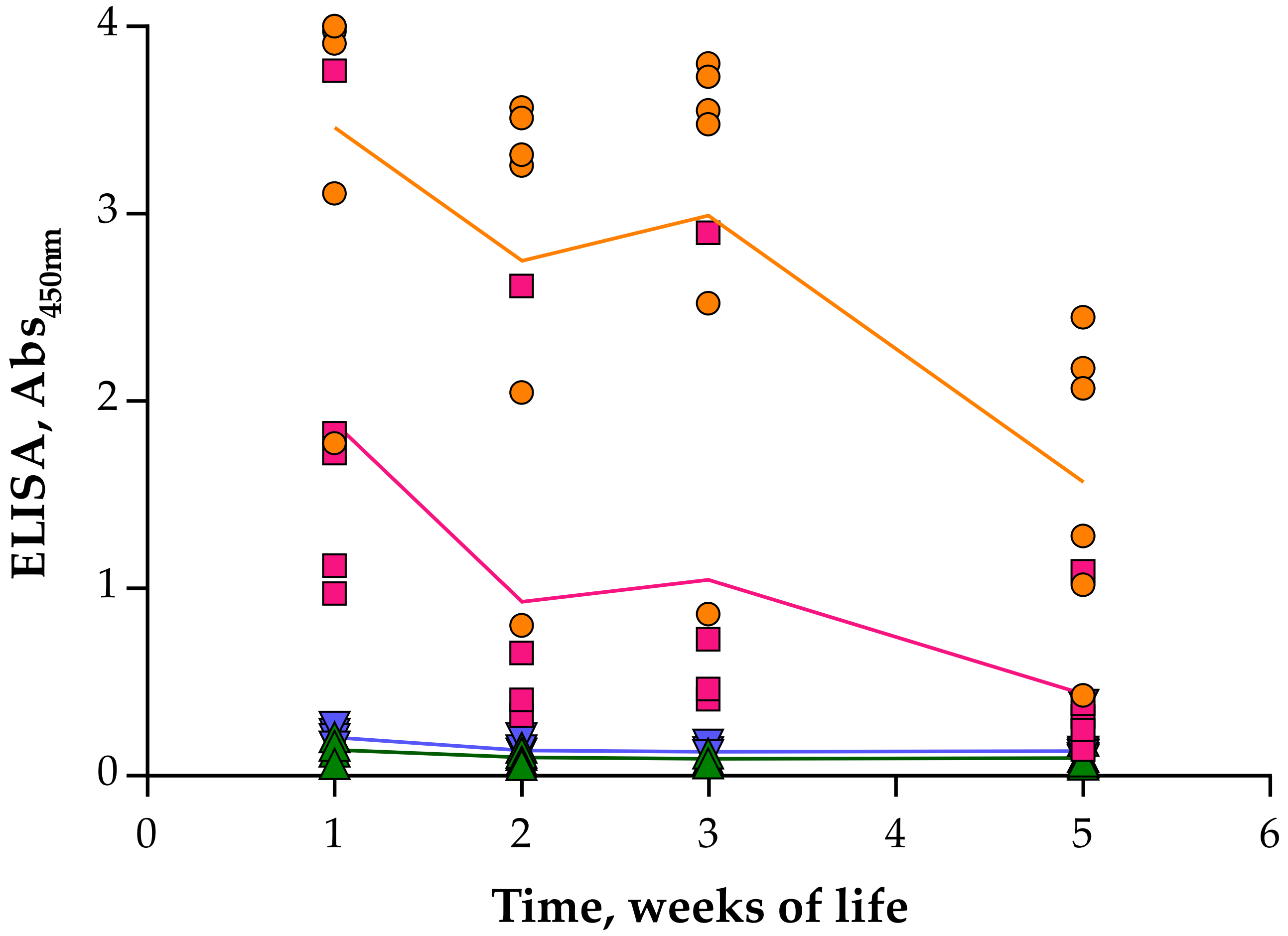

Figure S1. Levels of IgG against F4 in samples from individual piglets. Piglets from two vaccinated sows (vaccinated with F4 at 4 and 2 weeks before farrowing; pink and orange symbols and lines) and from two non-vaccinated sows were studied (blue and green symbols and lines). Serum samples from the piglets were taken at 1, 2, 3 and 5 weeks of life and tested for F4 antibodies by ELISA. At 22 days of life, piglets were challenged with *G. parasuis*. The average absorbance in the ELISA of the piglets from each biological sow is shown in the graph (lines).

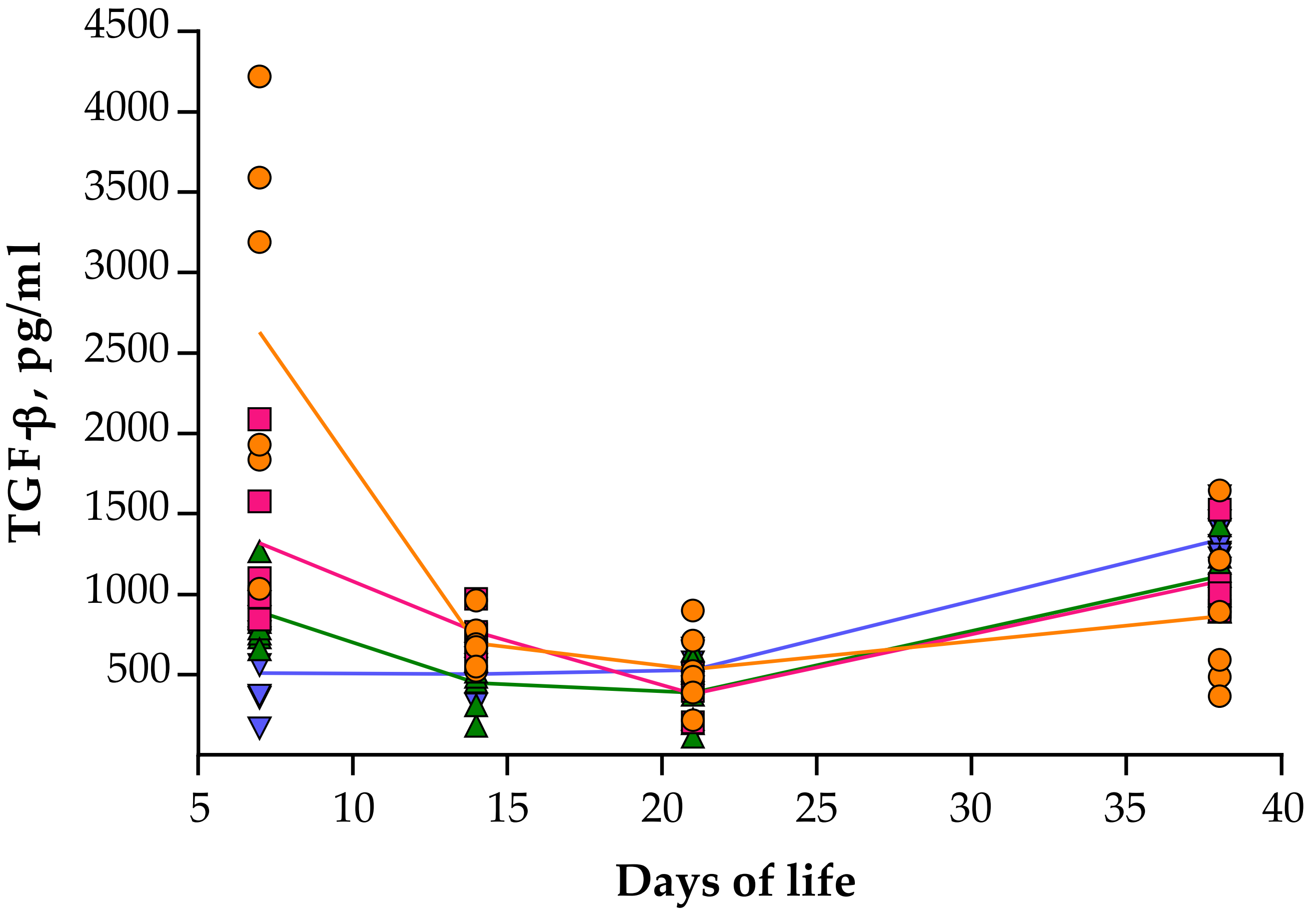

Figure S2. Levels of TGF-β in samples from individual piglets. Piglets from two vaccinated sows (vaccinated with F4 at 4 and 2 weeks before farrowing; pink and orange symbols and lines) and from two non-vaccinated sows were studied (blue and green symbols and lines). Serum samples from the piglets were taken at 1, 2, 3 and 5 weeks of life and the level of TGF-β was determined. At 22 days of life, piglets were challenged with *G. parasuis*. The average quantity of TGF-β of the piglets from each biological sow is shown in the graph (lines).

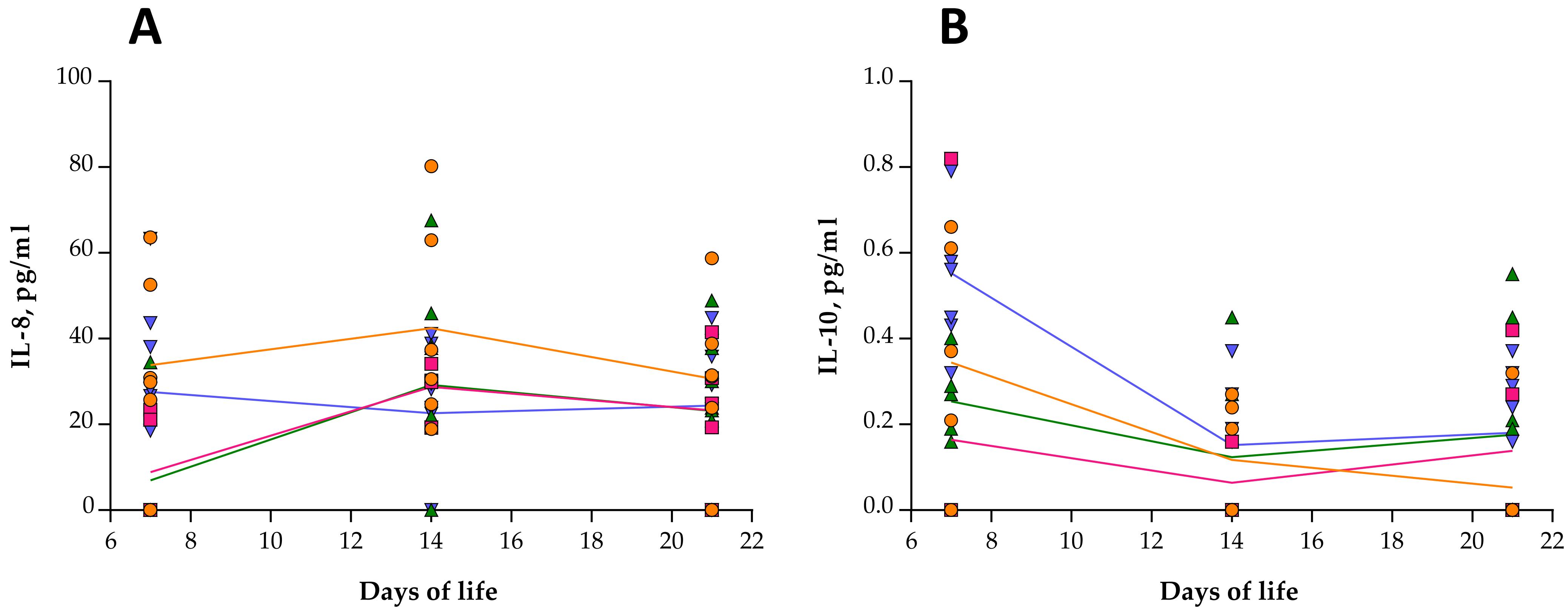

Figure S3. Levels of IL-8 (A) and IL-10 (B) in samples from individual piglets. Piglets from two vaccinated sows (vaccinated with F4 at 4 and 2 weeks before farrowing; pink and orange symbols and lines) and from two non-vaccinated sows were studied (blue and green symbols and lines). Serum samples from the piglets were taken at 7, 15, 21 days of life and the levels of IL-8 (A) and IL-10 (B) were determined. The average quantity of IL-8 (A) and IL-10 (B) of the piglets from each biological sow is shown in the graph (lines).

Table S1. Primers used in the study for detection of *Glaesserella parasuis*

Primer	Sequence (5′-3′)	Description
AV1-F	AAATATTTAGAGTTATTTGGAGTC	Detection of virulent and
V1-R	AATATACCTAGTAATACTAGACTTAAAAG	non-virulent strains[a]
NV1-R	CAGAATAAGCAAAATCAGC	
wcwK-F	CCACTGGATAGAGAGTGGCAGG	Detection of serovar 5/12
wcwK-R	CCATACATCTGAATTCCTAAGC	strains[b]
gltP-F	GCTGGAGGAGTTGAAAGAGTTGTTAC	Detection of serovar 13
gltP-R	CAATCAAATGAAACAACAGGAAGC	strains[b]

Galofré-Milà, N.; Correa-Fiz, F.; Lacouture, S.; Gottschalk, M.; Strutzberg-Minder, K.; Bensaid, A.; Pina-Pedrero, S.; Aragon, V. A Robust PCR for the differentiation of potential virulent strains of *Haemophilus parasuis*. BMC Vet. Res. 2017, 13, 1–5, doi:10.1186/s12917-017-1041-4.

[b] Howell, K.J.; Peters, S.E.; Wang, J.; Hernandez-Garcia, J.; Weinert, L.A.; Luan, S.L.; Chaudhuri, R.R.; Angen, Ø.; Aragon, V.; Williamson, S.M.; et al. Development of a multiplex PCR assay for rapid molecular serotyping of *Haemophilus parasuis*. J. Clin. Microbiol. 2015, 53, 3812–3821, doi:10.1128/JCM.01991-15
